# A risk of serious anaphylatic reactions to asthma biologics: a pharmacovigilance study based on a global real-world database

**DOI:** 10.1038/s41598-023-44973-z

**Published:** 2023-10-17

**Authors:** Sunny Park, Yeju Kim, Geon Ho Lee, Soo An Choi

**Affiliations:** 1 College of Pharmacy and Research Institute of Pharmaceutical Sciences, Korea University, Sejong, South Korea; 2 College of Pharmacy, Korea University, Sejong, South Korea

**Keywords:** Health care, Medical research, Signs and symptoms

## Abstract

Asthma is a chronic inflammatory condition that affects the lung airways. Chronic use of oral glucocorticoids in patients with severe asthma is associated with several adverse events (AEs). Biologics (omalizumab, benralizumab, mepolizumab, reslizumab, and dupilumab) have been developed as alternative therapies for the treatment of asthma. In this study, we aimed to evaluate the risk of anaphylactic reactions associated with these five biologics based on a large global database. We utilized individual case reports from the Uppsala Monitoring Center from January 1968 to December 29, 2019. A disproportionality analysis was performed over all drugs and monoclonal antibodies. Anaphylactic reactions were defined according to the "anaphylactic reaction” of the standardized MedDRA queries. Contrary to dupilumab, omalizumab, benralizumab, and mepolizumab demonstrated positive signals related to anaphylactic reactions over all drugs and monoclonal antibodies. Reslizumab, which represented only 315 cases of all AEs, requires more reports to determine its association with anaphylactic reactions. More anaphylactic reactions have been identified than are known, and most cases (96.2%) are reported to be serious. Our findings indicate that omalizumab, benralizumab, and mepolizumab for asthma treatment are associated with a high risk of anaphylactic reactions; thus, more careful monitoring in the post-administration period is recommended.

## Introduction

Asthma is a chronic inflammatory condition that affects the lung airways^1^, and it is a common respiratory disease that affects 350 million people worldwide^2^ and its global prevalence is increasing^3^. Asthma is associated with several comorbidities, including rhinitis, sinusitis, gastroesophageal reflux disease, and obstructive sleep apnea^4^. Patients with severe asthma require high doses of inhaled corticosteroids as well as the need for a second controller with or without systemic corticosteroids^5^. They experience increased hospitalization, a poor quality of life, and multiple adverse events (AEs) due to the chronic use of oral corticosteroids^6^. Systemic steroid use has several known side effects, including hyperglycemia, osteoporosis, adrenal suppression, dyslipidemia, cardiovascular disease, and Cushing’s syndrome, when used at high doses for prolonged periods^7^. Additionally, a few patients with severe asthma have a poor response to steroids, which complicates treatment^8^.

Biologics have been developed for asthma as alternative therapies to corticosteroids. They target receptors and cytokines involved in inflammatory pathogenesis^9^. Omalizumab, a monoclonal antibody (mAb) that inhibits immunoglobulin E (IgE)-mediated inflammation, was first approved by the Food and Drug Administration (FDA) for patients with asthma in 2003^10^. Several mAbs, including benralizumab, mepolizumab, reslizumab, and dupilumab, have entered the market as treatments for severe asthma.

Biologics used for asthma treatment are considered to have a relatively favorable safety profile; however, safety concerns have emerged with increased pharmacovigilance data gathered on mAbs for asthma^11,12^. AEs caused by biologics associated with anaphylactic reactions are rare, potentially severe, and sometimes life-threatening^13^. The incidence of anaphylaxis was < 0.1% and < 0.3% in pre-marketing clinical trials of omalizumab and reslizumab, respectively^14^. However, following its approval, the FDA issued a boxed warning of the anaphylaxis risk associated with omalizumab in 2007^15^. In a previous study, post-marketing safety evaluation has consistently raised the issue of anaphylactic reactions associated with biologics for asthma^16^. Furthermore, there is no study on the anaphylactic risk associated with biologics for treating asthma based on a large, global, real-world database. Therefore, in this study, we investigated the anaphylactic reaction risk associated with asthma biologics based on a large global database.

## Results

### Demographic characteristics of safety reports

Of the 21,161,249 reports covering all drugs, 62,883 (0.3%) reports identified the five biologics. Of the 62,883 reports, 1964 (3.1%) were anaphylactic reaction-related AEs. Overall, AEs and anaphylactic reaction reports were predominant among the American population and adult females. Although consumers or non-healthcare professionals reported most AEs, physicians were the most frequent reporters of anaphylactic reactions. Among the five biologics, omalizumab was the most frequently reported drug, followed by dupilumab (Table 1).

**Table 1:** Demographics of the total and anaphylactic reaction-related reports of asthma biologics on Vigibase.

Demographics	Total reports (N = 62,883)	Anaphylactic reaction reports (N = 1964)
Sex (N, %)		
Male	19,617 (31.2%)	304 (15.5%)
Female	38,276 (60.9%)	1410 (71.8%)
Not known	4990 (7.9%)	250 (12.7%)
Age		
< 18 years	2539 (4.0%)	137 (7.0%)
18–44 years	11,941 (19.0%)	508 (25.9%)
45–64 years	14,541 (23.1%)	339 (17.3%)
65–74 years	4213 (6.7%)	48 (2.4%)
≥ 75 years	1932 (3.1%)	17 (0.9%)
Unknown	27,717 (44.1%)	915 (46.6%)
Reporter		
Consumer/non-healthcare professional	29,155 (46.4%)	403 (20.5%)
Physician	20,083 (31.9%)	1,063 (54.1%)
Other healthcare professional	9707 (15.4%)	378 (19.3%)
Pharmacist	2373 (3.8%)	36 (1.8%)
Lawyer	11 (0.02%)	1 (0.05%)
Unknown	1554 (2.5%)	83 (4.2%)
Serious cases	22,995 (36.6%)	1,889 (96.2%)
Deaths	1441 (2.3%)	16 (0.8%)
Continent of the primary source		
Americas	50,837 (80.8%)	1565 (79.7%)
Europe	9656 (15.4%)	240 (12.2%)
Asia	1675 (2.7%)	88 (4.5%)
Oceania	573 (0.9%)	63 (3.2%)
Africa	142 (0.2%)	8 (0.4%)
Year		
≤ 2015	10,013 (15.9%)	897 (45.7%)
2016	4488 (7.1%)	204 (10.4%)
2017	9146 (14.5%)	207 (10.6%)
2018	12,845 (20.4%)	277 (14.1%)
2019	26,391 (42.0%)	379 (19.3%)
Condition^a^		
Asthma	24,868 (33.1%)	996 (41.5%)
Urticaria	7124 (9.5%)	396 (16.5%)
Dermatitis	12,392 (16.5%)	21 (0.9%)
Others	4464 (5.9%)	143 (6.0%)
Unknown	26,290 (35.0%)	847 (35.3%)
Drugs^b^		
Omalizumab	32,618 (51.6%)	1,760 (88.6%)
Mepolizumab	7344 (11.6%)	103 (5.2%)
Benralizumab	2387 (3.8%)	67 (3.4%)
Reslizumab	315 (0.5%)	5 (0.3%)
Dupilumab	20,559 (32.5%)	51 (2.6%)

^a^One case that reported one or more conditions.

^b^One patient that reported one or more drugs and AEs. The cases included suspect, concomitant, and interacting reports.

### Serious cases associated with anaphylactic reactions to asthma biologics

Most cases of anaphylactic reactions were serious (96.2%). As listed in Table 2, 1889 serious cases were associated with asthma biologics. Even with missing data, our study found that one-fifth of anaphylactic reaction cases resulted in prolonged hospitalization. The majority of cases show that the anaphylactic reaction has been disappeared upon the withdrawal of drug.

**Table 2:** Serious cases associated with anaphylactic reactions caused by asthma biologics.

N (%)	Omalizumab	Mepolizumab	Benralizumab	Reslizumab	Dupilumab
Seriousness^a^					
Caused/prolonged hospitalization	433 (21.9%)	28 (19.9%)	20 (23.8%)	2 (28.6%)	17 (27.0%)
Life-threatening	252 (12.8%)	19 (13.5%)	7 (8.3%)	1 (14.3%)	3 (4.8%)
Death	12 (0.6%)	3 (2.1%)	1 (1.2%)	0 (0%)	0 (0%)
Disabling/Incapacitating	10 (0.5%)	0 (0%)	0 (0%)	0 (0%)	0 (0%)
Congenital anomaly/Birth defect	1 (0.1%)	0 (0%)	0 (0%)	0 (0%)	0 (0%)
Other	1,255 (63.6%)	91 (64.5%)	56 (66.7%)	4 (57.1%)	43 (68.3%)
Unknown	10 (0.5%)	0 (0%)	0 (0%)	0 (0%)	0 (0%)
Outcome of serious AEs associated with anaphylactic reactions					
Recovered	599 (35.4%)	43 (43.4%)	27 (40.3%)	3 (75.0%)	13 (26.0%)
Recovering	78 (4.6%)	3 (3.0%)	1 (1.5%)	0 (0%)	5 (10.0%)
Recovered with sequelae	17 (1.0%)	0 (0%)	0 (0%)	0 (0%)	1 (2.0%)
Not recovered	29 (1.7%)	4 (4.0%)	1 (1.5%)	0 (0%)	0 (0%)
Fetal	6 (0.4%)	0 (0%)	1 (1.5%)	0 (0%)	0 (0%)
Unknown	962 (56.9%)	49 (49.5%)	37 (55.2%)	1 (25.0%)	31 (62.0%)
Actions taken to address AEs					
Drug withdrawn	646 (38.2%)	40 (40.4%)	23 (34.3%)	3 (75.0%)	14 (28.0%)
Dose not changed	90 (5.3%)	5 (5.1%)	8 (11.9%)	1 (25.0%)	9 (18.0%)
Dose reduced	14 (0.8%)	0 (0%)	0 (0%)	0 (0%)	0 (0%)
Dose increased	11 (0.7%)	0 (0%)	0 (0%)	0 (0%)	0 (0%)
Not applicable	20 (1.2%)	4 (4.0%)	2 (3.0%)	0 (0%)	0 (0%)
Unknown	910 (53.8%)	50 (50.5%)	34 (50.8%)	0 (0%)	27 (54.0%)
Outcomes after actions					
Reaction abated	692 (40.9%)	43 (43.4%)	25 (37.3%)	3 (75.0%)	19 (38.0%)
No effect observed	28 (1.7%)	4 (4.0%)	1 (1.5%)	0 (0%)	0 (0%)
Effect unknown	966 (57.1%)	52 (52.5%)	40 (59.7%)	1 (25.0%)	31 (62.0%)
Fetal	5 (0.3%)	0 (0%)	1 (1.5%)	0 (0%)	0 (0%)

Serious cases included reports related to anaphylactic reactions regardless of positive signals.

^a^Cases reported with one or more than two kinds of seriousness.

### Disproportionality analysis

Omalizumab, benralizumab, and mepolizumab demonstrated positive signals for anaphylactic risk over all drugs and mAbs. Two-track analysis showed similar results, and various anaphylactic reaction terms were detected over all mAbs for omalizumab. The number of reports was relatively small, and no signal was detected for reslizumab. Additionally, no positive signals related to anaphylactic reactions were observed for dupilumab. Table 3 lists all AEs associated with anaphylactic reactions reported for the five biologics and the results of the disproportionality analysis.

**Table 3:** Disproportionality analysis of outcomes associated with anaphylactic reactions.

Drugs (total no. of reports)	Anaphylactic reaction associated AEs^a^	No. of reports (%)	PRR	ROR	IC_025_
Omalizumab (32,457)	Anaphylactic reaction^b^	1,437 (4.4%)	9.61	10.01	3.17
	Anaphylactic shock	193 (0.6%)	1.51	1.51	0.38
	Anaphylactoid reaction	82 (0.3%)	1.05	1.05	− 0.26
	Anaphylactoid shock	2 (< 0.1%)	1.74	1.74	− 1.99
	Circulatory collapse	12 (< 0.1%)	0.30	0.30	− 2.63
	Kounis syndrome	1 (< 0.1%)	1.34	1.35	− 3.53
	Shock	12 (< 0.1%)	0.51	0.51	− 1.88
	Shock symptom	1 (< 0.1%)	0.91	0.91	− 3.89
	Type I hypersensitivity^b^	34 (0.10%)	6.12	6.12	1.97
Mepolizumab (7283)	Anaphylactic reaction^b^	84 (1.2%)	2.47	2.49	0.97
	Anaphylactic shock	11 (0.2%)	0.38	0.38	− 2.32
	Anaphylactoid reaction	5 (< 0.1%)	0.29	0.29	− 3.24
	Circulatory collapse	2 (< 0.1%)	0.22	0.22	− 2.63
	Shock	1 (< 0.1%)	0.19	0.19	− 5.75
	Type I hypersensitivity	1 (< 0.1%)	0.80	0.80	− 4.03
Benralizumab (2363)	Anaphylactic reaction^b^	54 (2.3%)	4.90	4.99	1.83
	Anaphylactic shock	3 (0.1%)	0.32	0.32	− 3.54
	Anaphylactoid reaction	3 (0.1%)	0.53	0.53	− 2.87
	Circulatory collapse	2 (0.1%)	0.68	0.68	− 3.04
	Kounis syndrome	1 (< 0.1%)	18.50	18.51	− 2.36
	Shock	1 (< 0.1%)	0.58	0.58	− 4.37
	Type I hypersensitivity	1 (< 0.1%)	2.45	2.45	− 3.07
Reslizumab (313)	Anaphylactic reaction	4 (1.3%)	2.74	2.76	− 0.54
	Anaphylactoid reaction	1 (0.3%)	1.33	1.33	− 3.54
Dupilumab (20,548)	Anaphylactic reaction	37 (0.2%)	0.39	0.38	− 1.86
	Anaphylactic shock	4 (< 0.1%)	0.05	0.05	− 5.92
	Anaphylactoid reaction	1 (< 0.1%)	0.02	0.02	− 8.85
	Circulatory collapse	4 (< 0.1%)	0.16	0.16	− 4.26
	Shock	2 (< 0.1%)	0.13	0.13	− 5.22

PRR, Proportional reporting ratio; ROR, Reporting odds ratio; IC, information component; IC_025_, under 95% confidence interval of IC.

^a^Anaphylactic reaction-related AEs were selected using the “anaphylactic reaction” of the standardized MedDRA Query (SMQ).

^b^Positive signals detected by disproportionality analysis.

## Discussion

Based on a global real-world database, this study evaluated the risk of anaphylactic reactions associated with five biologics for asthma treatment. We identified that omalizumab, mepolizumab, and benralizumab were associated with a serious anaphylactic reactions. In particular, omalizumab had a relatively high risk of anaphylaxis with a higher disproportionality index than in the other biologics. In a previous study, it was reported that omalizumab was associated with 0.1–0.2% of anaphylaxis incidence^17^. It was much less compared to our results. Mepolizumab and benralizumab also have a risk of anaphylactic reactions^18,19^, which is consistent with our findings. Regarding reslizumab, the incidence of anaphylaxis in clinical trials was approximately 0.3% but there were only 315 cases of total AEs; therefore, more reports were required to determine the association with anaphylactic reactions^14^. Unlike other biologics, dupilumab showed no signal of anaphylactic reactions despite the large number of AEs reported, which is consistent with a previous study^20^.

Above all, this is the first study to analyze the risk of anaphylactic reactions for five biologics, not only over all drugs but also over other mAbs. The result showed that omalizumab, benralizumab, and mepolizumab have more apparent anaphylactic reactions than all other drugs, especially mAbs. Although biologics are known to induce anaphylaxis^21^, these three biologics have a disproportionately high number of reported anaphylactic reactions, implying an association with anaphylaxis as compared to other mAbs. Therefore, more careful monitoring of anaphylactic reactions following asthma treatment with omalizumab, benralizumab, and mepolizumab is necessary than in other mAbs.

A possible explanation for biologic-induced anaphylactic reactions is anti-drug antibodies (ADAs), which are considered to be the primary inducers of these reactions to biologics^21^. However, a meta-analysis study reported the highest and lowest amounts of ADAs in benralizumab (8.35%) and omalizumab (0.00%), respectively^22^. In addition, the incidence of ADA in dupilumab studies was 7.61%^22^, which is inconsistent with our results. Another potential cause of anaphylaxis is polysorbate, which is one of the excipients^23^. A case study of anaphylaxis after omalizumab administration revealed polysorbate positivity in the absence of IgE and IgG antibodies for omalizumab^24^. Omalizumab and benralizumab contain polysorbate 20, and mepolizumab and dupilumab contain polysorbate 80. In contrast, reslizumab does not contain any polysorbates^25^. Both polysorbate 20 and 80 are inducers of anaphylactic reactions, but they have no clear differences^24^. Therefore, polysorbate can not be fully accountable for our results based on real-world data, as dupilumab showed no association with anaphylactic reactions. Overall, the degree of humanization is most likely the reason for anaphylaxis; dupilumab is a fully humanized mAb with a 99% human component, whereas other biologics have a 90% human component, as reported in a previous study^20^, which is consistent with our results.

Anaphylaxis is a systemic, possibly life-threatening condition^26^, which highlights the importance of our research. Anaphylactic reactions are considered as not being serious at times^14^. However, in our study, anaphylactic reactions to biologics were found to have a higher proportion of serious cases (96.2%) than in all AEs (36.6%). Actually, other study similarly reported serious cases of mAb-related all AEs (30.3%)^27^. In 2007, an omalizumab joint task force was established; they recommended a post-injection observation period after omalizumab administration^28^. Approximately 77% of anaphylactic reactions to omalizumab were reported at a medical facility^17^, which is consistent with our finding that most anaphylactic reactions were reported by physicians, whereas most AEs were reported by consumers. Although death events accounted for only 0.8% of serious anaphylactic reactions in this study, approximately 2% were fatal or did not fully recover, resulting in an overall health and economic burden. With the exception of missing cases, the drug was discontinued in majority of the cases, and the reaction was abated, supporting drug-induced anaphylactic reactions. Therefore, anaphylactic reactions to biologics are reported to be more serious in the real world than is known in studies, so more careful monitoring is needed, which will contribute to preventing mAb treatment failure.

Previous studies reported that AEs and anaphylactic reactions predominantly occurred in adult females. However, the incidence could not be estimated^20,29,30^. A higher incidence of anaphylaxis was reported in South Asian populations than in Caucasian ones^31^, and a slight increase in the proportion of anaphylaxis compared to that with all AEs was observed in Asians. However, further studies are required to investigate ethnic differences.

This study used a database of spontaneous reports; therefore, there were limitations of under- and over-reporting, and it was not possible to estimate the incidence rate of anaphylactic reactions. However, this study is valuable in several respects. We used a global real-world database to obtain a global perspective on AEs and anaphylactic reaction reports. Also, this study has an advantage over a previous study^20^ in that it expands the terminology by using standardized MedDRA queries to define anaphylactic reaction terms. These results are noteworthy because anaphylactic reactions can be severe and/or life-threatening. In conlclusion, our findings suggest that omalizumab, benralizumab, and mepolizumab have a high risk of serious anaphylactic reactions and more careful monitoring in the post-injection period is recommended.

## Methods

### Data source

Individual case safety reports (ICSRs) from the World Health Organization Uppsala Monitoring Center (WHO-UMC) Vigibase of Biologics for asthma (omalizumab, mepolizumab, reslizumab, benralizumab, and dupilumab) were used in this study. The data included information on age group, sex, reporter, date, continent of the primary source, name of drug used, AEs, and seriousness reported by members participating in the WHO International Drug Monitoring Program from 1968 to December 29, 2019. ICSRs were received from local physicians, pharmacists, other healthcare providers, and the public. The US FDA defined serious AEs as those that resulted in death, a life-threatening condition, hospitalization (initial or prolonged), disability or permanent damage, a congenital anomaly or birth defect, and requiring intervention to prevent permanent impairment or damage^32^. We analyzed all and anaphylactic reaction related ICSRs of 5 biologics. Given that anaphylactic reactions are systemic, life-threatening^26^, outcomes and interventions after drug administration were examined in serious cases. This study was approved by the Institutional Review Board (IRB) of Korea University, which waived the requirement for informed consent due to the use of secondary data (IRB No. 2020–0208). All research procedures were performed in accordance with the relevant guidelines and regulations.

### Data mining and signal detection criteria

A two-by-two table was used to investigate the disproportionality, a method used as a basic approach for detecting signals in large databases (Table 4). The most frequently used disproportionality parameters, proportional reporting ratio (PRR), reporting odds ratio (ROR), and information component (IC)^33,34^, were calculated based on the cases reported as suspicious or interacting.

**Table 4:** Two-by-two contingency table for disproportionality analysis.

Number of reports	Interest AEs	All other AEs
Drug of interest	A	B
All other drugs (or all mAbs)	C	D

The number of reports included in A: both target drugs and specific AEs; B: target drug AEs but with all other AEs; C: specific AEs but with all other drugs; D: all other drugs and all other AEs.

Because the anaphylactic reactions of mAbs are well known^35^, disproportionality analysis was performed over all drugs, and all reported mAbs (Supplementary information 1). For events reported at least three times, positive signals were defined when the PRR and ROR were ≥ 2 and below the IC limit of 95% ≥ 0, as shown in Table 5.

**Table 5:** Formulae and criteria for signal detection.

Indices	Formula	Signal detection criteria
PRR	[A/(A + B)]/[C/(C + D)]	PRR ≥ 2
ROR	(A/B)/(C/D)	ROR ≥ 2
IC	IC = log_2_P(AE, Drug)/P(AE)P(Drug)	Under a limit of 95% CI ≥ 0

RRR, proportional reporting ratio; ROR, reporting odds ratio; IC, information component; AE, adverse event; CI, confidence interval.

### Standardized Medical Dictionary for Regulatory Activities (MedDRA) Query (SMQ) and the definition of an anaphylactic reaction

The MedDRA terminology, the global standard for recording AEs and medical histories^36^, was used to obtain data. It has five hierarchical structures: system organ class, high-level group term, high-level term, preferred term (PT), and lowest-level term^37^. The disproportionality analysis was conducted on the PT level. The SMQ, a validated and pre-determined set of MedDRA terms^38^, was used to group anaphylactic reaction terms. Our study defined anaphylactic reactions as “anaphylactic reactions” of the SMQ in a narrow scope, including, “anaphylactic reaction,” “anaphylactic shock,” “anaphylactic transfusion reaction,” “anaphylactoid reaction,” “circulatory collapse,” “Kounis syndrome,” “procedural shock,” “shock,” “shock syndrome,” and “type 1 hypersensitivity” for their PTs.

### Ethics declarations

The study was approved by Korea University’s Institutional Review Board (IRB No. 2020–0208).

### Supplementary Information

Supplementary Information.

## Data Availability

The datasets analyzed are not publicly available because of the ongoing collection of AE reports. However, they are available from UMC upon reasonable request. Data will be available after obtained approval from the UMC at https://who-umc.org/ (request number ER198-2019).
