# Supplementary material for: A risk of serious anaphylatic reactions to asthma biologics: a pharmacovigilance study based on a global real-world database
Source: Sci Rep. 2023 Oct 17;13:17607. doi: 10.1038/s41598-023-44973-z (PMC10582024; doi:10.1038/s41598-023-44973-z)
Supplement: Supplementary file 1 — Supplementary Information. [file 41598_2023_44973_MOESM1_ESM.docx]

Supplementary information 1. The list of all monoclonal antibodies reported to Vigibase (216).

| Abagovomab |
| --- |
| Abciximab |
| Abituzumab |
| Adalimumab |
| Aducanumab |
| Alemtuzumab |
| Alirocumab |
| Amatuximab |
| Andecaliximab |
| Anetumab ravtansine |
| Anifrolumab |
| Atezolizumab |
| Avelumab |
| Bapineuzumab |
| Basiliximab |
| Bavituximab |
| Begelomab |
| Belimumab |
| Benralizumab |
| Besilesomab |
| Bevacizumab |
| Bezlotoxumab |
| Bimagrumab |
| Bimekizumab |
| Bleselumab |
| Blinatumomab |
| Bococizumab |
| Brentuximab vedotin |
| Briakinumab |
| Brodalumab |
| Brolucizumab |
| Budigalimab |
| Burosumab |
| Cabiralizumab |
| Canakinumab |
| Caplacizumab |
| Carotuximab |
| Catumaxomab |
| Cemiplimab |
| Cergutuzumab amunaleukin |
| Certolizumab |
| Certolizumab pegol |
| Cetuximab |
| Cixutumumab |
| Clazakizumab |
| Codrituzumab |
| Conatumumab |
| Crenezumab |
| Dacetuzumab |
| Daclizumab |
| Dalotuzumab |
| Daratumumab |
| Demcizumab |
| Denosumab |
| Depatuxizumab |
| Depatuxizumab mafodotin |
| Dinutuximab |
| Dinutuximab beta |
| Duligotumab |
| Dupilumab |
| Durvalumab |
| Eculizumab |
| Edrecolomab |
| Efalizumab |
| Efungumab |
| Eldelumab |
| Elgemtumab |
| Elotuzumab |
| Emactuzumab |
| Emapalumab |
| Emibetuzumab |
| Emicizumab |
| Enfortumab vedotin |
| Enoblituzumab |
| Epratuzumab |
| Erenumab |
| Etaracizumab |
| Etrolizumab |
| Evolocumab |
| Farletuzumab |
| Ficlatuzumab |
| Figitumumab |
| Fremanezumab |
| Fresolimumab |
| Galcanezumab |
| Galiximab |
| Ganitumab |
| Gemtuzumab |
| Gevokizumab |
| Girentuximab |
| Glembatumumab |
| Golimumab |
| Guselkumab |
| Ianalumab |
| Ibalizumab |
| Ibritumomab tiuxetan |
| Icrucumab |
| Idarucizumab |
| Indatuximab ravtansine |
| Indium (111 in) ibritumomab tiuxetan |
| Infliximab |
| Inolimomab |
| Inotuzumab |
| Intetumumab |
| Ipilimumab |
| Isatuximab |
| Iscalimab |
| Itolizumab |
| Ixekizumab |
| Keliximab |
| Lanadelumab |
| Lebrikizumab |
| Lintuzumab |
| Lirilumab |
| Lorvotuzumab mertansine |
| Lumiliximab |
| Lumretuzumab |
| Lutikizumab |
| Mapatumumab |
| Matuzumab |
| Mepolizumab |
| Mirikizumab |
| Mirvetuximab soravtansine |
| Mogamulizumab |
| Mosunetuzumab |
| Motavizumab |
| Moxetumomab pasudotox |
| Naptumomab Estafenatox |
| Natalizumab |
| Nebacumab |
| Necitumumab |
| Nesvacumab |
| Nimotuzumab |
| Nivolumab |
| Obinutuzumab |
| Ocrelizumab |
| Ofatumumab |
| Olaratumab |
| Oleclumab |
| Omalizumab |
| Onartuzumab |
| Ontuxizumab |
| Opicinumab |
| Oregovomab |
| Otelixizumab |
| Otlertuzumab |
| Palivizumab |
| Pamrevlumab |
| Panitumumab |
| Pasotuxizumab |
| Patritumab |
| Pembrolizumab |
| Pertuzumab |
| Pidilizumab |
| Polatuzumab vedotin |
| Racotumomab |
| Ramucirumab |
| Ranibizumab |
| Ravulizumab |
| Raxibacumab |
| Reslizumab |
| Rilotumumab |
| Rinucumab |
| Risankizumab |
| Rituximab |
| Romosozumab |
| Rovalpituzumab tesirine |
| Sarilumab |
| Satumomab pendetide |
| Secukinumab |
| Selicrelumab |
| Seribantumab |
| Siltuximab |
| Simtuzumab |
| Sirukumab |
| Solanezumab |
| Spartalizumab |
| Tabalumab |
| Talacotuzumab |
| Tanezumab |
| Tarextumab |
| Technetium (99Mtc) Fanolesomab |
| Technetium sulesomab (99mTc) |
| Technetium-99m arcitumomab |
| Telisotuzumab vedotin |
| Teprotumumab |
| Tesidolumab |
| Tezepelumab |
| Tigatuzumab |
| Tildrakizumab |
| Tisotumab vedotin |
| Tocilizumab |
| Tositumomab |
| Tralokinumab |
| Trastuzumab |
| Trastuzumab duocarmazine |
| Trastuzumab emtansine |
| Tremelimumab |
| Ublituximab |
| Ulocuplumab |
| Urelumab |
| Ustekinumab |
| Utomilumab |
| Vadastuximab talirine |
| Vantictumab |
| Vanucizumab |
| Varlilumab |
| Vedolizumab |
| Visilizumab |
| Vobarilizumab |
| Volociximab |
| Vonlerolizumab |
| Xentuzumab |
| Yttrium 90 Ibritumomab Tiuxetan |
| Zalutumumab |
| Zanolimumab |
